# Women in larger bodies’ experiences with contraception: a scoping review

**DOI:** 10.1186/s12978-021-01139-2

**Published:** 2021-04-29

**Authors:** Tierney M. Boyce, Elena Neiterman

**Affiliations:** School of Public Health and Health Systems, Faculty of Health, University of Waterloo, 200 University Avenue West, Waterloo, ON N2L 3G1 Canada

**Keywords:** Contraception, Emergency contraception, Overweight, Obesity, Body Mass Index, Sexual and Reproductive Health, Scoping review

## Abstract

**Background:**

As the prevalence of obesity increases and the age of onset decreases, more women of reproductive age will be living in larger bodies. Research on weight-related efficacy and safety has informed clinical guidelines for routine and emergency contraceptive use by women with a higher body mass index; however, patient perspectives are needed to understand women in larger bodies’ experiences with contraception and contraceptive care. This scoping review summarizes the literature on women in larger bodies’ experiences with contraception with the goal of gaining a better understanding of the nature of these experiences and identifying gaps in the existing research.

**Methods:**

Following Arksey and O’Malley’s framework, a scoping review of the literature was conducted. Four databases (PubMed, PsycINFO, SCOPUS, and CINAHL) were searched for peer-reviewed, empirical articles published in English between 2010 and 2020, with a focus on North America, Europe, Australia, and New Zealand. Data were summarized by identifying key themes in the reviewed literature.

**Results:**

Twenty-nine articles meeting the eligibility criteria were reviewed. The literature was predominantly quantitative (n = 27), with only one qualitative study and one systematic review, respectively. Five themes were identified, including (1) use of contraception among women in larger bodies; (2) knowledge, attitudes towards and beliefs about contraception; (3) contraceptive (dis)satisfaction among women in larger bodies; (4) contraceptive counseling; and (5) barriers to contraception. The findings revealed that women in larger bodies may have unmet contraceptive care needs. Despite many articles addressing the need to improve contraceptive counseling for women in larger bodies (n = 26), few explored how women felt about their care (n = 2). Finally, only two articles focused on emergency contraception, indicating a need for further research.

**Conclusion:**

This scoping review emphasizes the pressing need for qualitative research to explore women in larger bodies’ experiences with routine and emergency contraception, as well as receiving contraceptive counseling and care. Future research exploring the lived experiences of women in larger bodies is necessary to better characterize their contraceptive needs and identify avenues to improve patient care.

**Supplementary Information:**

The online version contains supplementary material available at 10.1186/s12978-021-01139-2.

## Plain English summary

As obesity becomes more prevalent, more women of reproductive age will be living in larger bodies. This review highlights what is known about women in larger bodies’ experiences with contraception and suggests where future research is needed. We searched four databases for papers published in English from 2010 to 2020. Our review included 29 articles. Nearly all of the studies were quantitative, with one qualitative study and one review. Most articles talked about contraceptive use, but few of them included emergency contraception. Some papers highlighted women’s knowledge and opinions about contraception, whereas others talked about their contraceptive (dis)satisfaction. Many articles discussed contraceptive counseling. Finally, some papers considered potential barriers to contraception. Our findings suggest that women in larger bodies have unmet contraceptive needs. Notably, this review aimed to focus on patient experiences but women’s voices were rarely represented. Future qualitative research on women’s lived experiences will provide a better understanding of women in larger bodies’ contraceptive needs and suggest how care can be improved.

**Supplementary Information:**

The online version contains supplementary material available at 10.1186/s12978-021-01139-2.

## Introduction

The global prevalence of overweight and obesity has nearly tripled in the last four decades [1], resulting in what has become known as an ‘obesity epidemic’ [2]. In the United States, nearly half of women of reproductive age report a height and weight that is characterized as ‘overweight’ or ‘obese’ based on body mass index (BMI) classification [3, 4]. While obesity is not universally defined, body weight status is most commonly estimated by calculating BMI, a weight to height ratio that classifies ‘overweight’ as a BMI ≥ 25 kg/m^2^ and ‘obesity’ as a BMI ≥ 30 kg/m^2^ [5]. In addition to being a well-known risk factor for a number of chronic conditions, higher BMI may also have a marked impact on reproductive health [6].

Women’s sexual behavior does not seem to vary by body size [7, 8], and while some studies suggest that women with higher BMIs may experience reduced contraceptive efficacy [9, 10] and increased rates of unintended pregnancy [11], most research on contraception does not include women with higher BMIs [12]. The existing literature on this topic is limited and shows some inconsistencies in findings [9, 13, 14]; however, there is pharmacokinetic evidence demonstrating that some hormonal contraceptives, such as the transdermal patch and levonorgestrel (LNG) emergency contraception (EC), may have reduced efficacy when used by women with higher BMIs [9, 14]. In addition, BMI ≥ 30 and combined oral contraceptive (COC) use are both risk factors for venous thromboembolism, resulting in an elevated risk among women with higher BMIs who use COCs [15]. Such research findings have informed the World Health Organization’s medical eligibility criteria [16] and various clinical guidelines for contraceptive use in North America and Europe [17–20]. Moreover, warnings of reduced efficacy among users who weigh over 165 lbs (75 kg) and inefficacy among users who weigh over 175 lbs (80 kg) were introduced to Canadian LNG EC labels in 2014 [21] and briefly in Europe from 2013 to 2014 [22]. Other international organizations have suggested that women with higher BMIs may be offered a double dose of LNG when other EC options, such as ulipristal acetate (UPA) and the copper-bearing intrauterine device (Cu-IUD), are not feasible [20, 23]. Overall, efficacy and risk-based evidence indicate that progestin-only contraception, contraceptive injections, and long-acting reversible contraception (LARC), such as intrauterine devices (IUDs), intrauterine systems (IUSs) and contraceptive implants, are preferred for women with higher BMIs [13, 24].

Given that the benefits of using contraception outweigh the risks associated with non-use, women in larger bodies^1^ should be counseled on all methods of contraception to inform their choices [9, 29]. This is of particular relevance in the context of bariatric surgery, as the weight loss may increase fertility and the resulting nutritional deficiencies pose a significant risk to maternal and fetal health [30]. Pregnancy should be avoided for 1 to 2 years postoperatively and thus, contraceptive counseling plays an integral role in bariatric care for women of reproductive age [31]. Nonetheless, although contraceptive options should not be restricted solely on the basis of weight [29], the research forming the basis of weight-related contraceptive guidelines reveals little about women in larger bodies’ experiences.

Patient perspectives are needed to understand women in larger bodies’ experiences with contraception and contraceptive care. The focus of this scoping review is to answer the following research question: “What does academic literature reveal about women in larger bodies’ experiences with contraception?”. Ultimately, the aim of this scoping review is to better understand the contraceptive experiences of women in larger bodies, to identify gaps in the literature, and to provide recommendations for future research.

## Methods

This scoping review was guided by the methodological framework developed by Arksey and O’Malley [32]. This methodology was chosen to reflect our broad research objectives to examine the breadth and range of the current body of literature and identify areas where future research is needed [32]. Following Arksey and O’Malley’s framework, the following five steps were taken: (1) identifying the research question; (2) identifying relevant literature; (3) selecting literature based on the inclusion and exclusion criteria; (4) charting the data; and (5) collating, summarizing, and reporting the results [32].

### Identifying the research question

We defined the parameters of this scoping review by developing a research question that asked, “What does academic literature reveal about women in larger bodies’ experiences with contraception?”. This broad approach was taken to capture all relevant literature [32]. After an initial literature search, we identified specific sub-questions to provide a clear understanding of our research goals and to inform our search strategy:

1. What attitudes do women in larger bodies have about contraception?
2. What contraceptive decisions are made by women in larger bodies?
3. Do women in larger bodies receive contraceptive counseling?
4. What barriers do women in larger bodies face when seeking contraceptive care?

### Identifying relevant literature

From the above stated research questions, we established a search strategy in consultation with a professional librarian (see Table 1). First, a list of keywords related to our research questions was developed. Although we refer to ‘larger bodies’ throughout this paper, we chose body descriptor search terms like ‘overweight’ and ‘obese’ to reflect the dominant biomedical discourse which uses BMI classification to characterize body size [33]. We piloted our initial combination of terms, made adjustments through a trial-and-error process, and finalized our search strategy. On June 25, 2020, the search was performed in four databases: PubMed, SCOPUS, PsycINFO, and CINAHL. To collect up-to-date and relevant literature, we limited our search to papers published between 2010 and 2020 in peer-reviewed journals. We only included the literature published in English.

**Table 1:** Search strategy used for literature search

(contracepti* OR “birth control” OR “fertility control” OR preconception)
(obesity OR obese OR overweight OR “body mass index” OR BMI)
(attitude* OR knowledge OR perception* OR belief* OR use OR prefer* OR behavior* OR behaviour* OR decision* OR counsel* OR feel* OR access* OR seek* OR barrier* OR “focus group*” OR interview* OR qualitative OR experience*)

### Selecting the literature

**Fig. 1:**
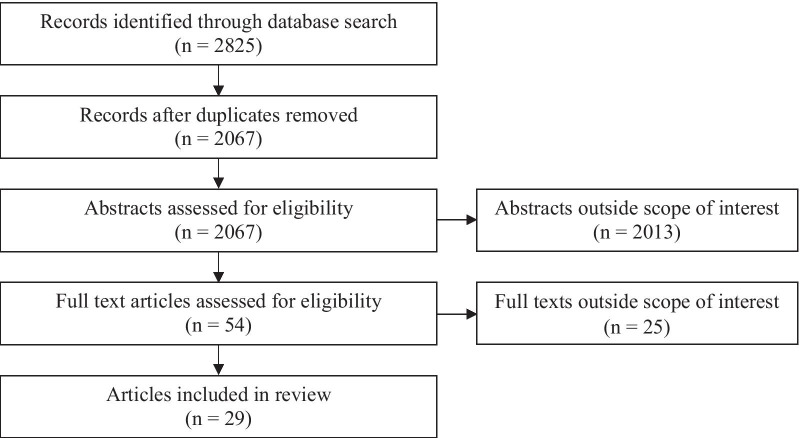
PRISMA flow diagram

The research results were imported into RefWorks, an online reference manager, where they were screened for eligibility. After removing duplicates, we screened the abstracts of the 2067 articles. To collect evidence-based information relevant to the health care and societal contexts of Western nations, we limited our review to empirical research, including systematic reviews, conducted in North America, Europe, Australia, and New Zealand. The focus on Western countries was selected to collect data from high-income countries with comparable health care systems [34–36], and to reflect the belief that Westernization has played a role in the increasing global prevalence of higher BMIs and related chronic conditions [37]. Studies that did not include women and articles without an analytic focus on contraception were excluded. Since the goal of the review was to capture women’s experiences with contraception, we excluded clinical trials and papers that focused on clinical efficacy or safety, incidence of adverse effects, and health care providers’ perspectives. Moreover, studies with a specified focus on the use of contraception for non-contraceptive medical purposes (e.g., polycystic ovarian syndrome, endometriosis, acne) were excluded. We included studies that focused on women undergoing bariatric surgery. Outside of this clinical context, we excluded studies that did not classify participants as ‘overweight’, ‘obese’ or by BMI, as well as articles that did not present results about body size. During this initial abstract screening, 2013 articles were excluded for being outside our scope of interest. We conducted a full-text screening of the remaining 54 articles, discussed any disagreements that arose with respect to eligibility, and reached a consensus to include 29 articles in the full review. The results from our literature search and selection process are outlined in Fig. 1.

### Charting the data

Using Microsoft Excel, a literature extraction tool was developed to record information about each article. This included authors, year of publication, region of research, study title and objectives, study methods, type of contraception, body size indication, and study sample demographics. Our analysis began with each author independently reading and carefully reviewing five articles to identify key themes in the research. We met to discuss our findings and prioritize the themes that would guide our analysis, in consideration of our a-priori research questions. After some refinement, we finalized the key themes that were applied to each article for the full review.

### Collating, summarizing, and reporting the results

Five key themes were added to the literature extraction tool, where data from each article was imported: (1) use of contraception among women in larger bodies; (2) knowledge, attitudes towards and beliefs about contraception; (3) contraceptive (dis)satisfaction among women in larger bodies; (4) contraceptive counseling; and (5) barriers to contraception. Many articles had data extracted for more than one theme; however, the relevancy of each theme to the overall article was considered throughout the analysis and major themes were prioritized in the findings.

## Results

### Characteristics of the reviewed literature

**Fig. 2:**
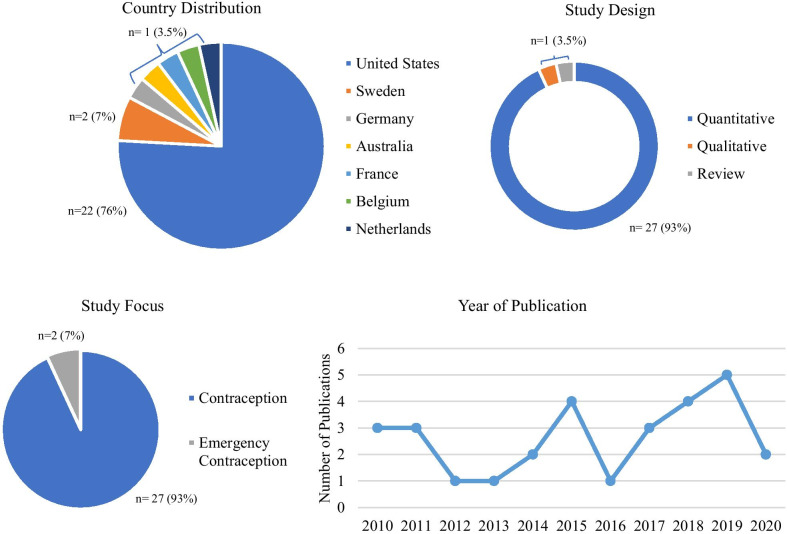
Study sample characteristics

Twenty-nine articles met the eligibility criteria. An overview of the literature demographics can be found in Fig. 2. The majority of the research was conducted in the United States (n = 22), while Swedish studies represented 7% of the articles (n = 2), and the rest of the research was conducted in Belgium, Netherlands, France, Germany, and Australia, each representing 3.5% (n = 1) of the total sample. Nearly half of the articles (n = 14) were published since 2017, suggesting a growing research interest in this area. The literature glaringly lacks qualitative inquiry, as the vast majority of the empirical literature included in this review was quantitative (n = 27), with only one study employing a qualitative design and one systematic review. Fewer than 10% of studies focused on emergency contraception (n = 2), indicating a need for future research.

Total study sample sizes ranged from 25 to 147 336 participants, with nearly half of the papers reporting results from samples of greater than 1000 participants (n = 14). Most studies reported a sample of participants who were predominantly white (n = 19). Notably, 24% of the articles did not provide information on participants’ race or ethnicity (n = 7). Although some studies had a broader sexual or reproductive health focus (n = 8), all data on contraception was limited to participants of peri-reproductive age, with study samples including girls and women between the ages of 13 and 49 years old. While there was a significant research focus on patients who underwent bariatric surgery (n = 13), a little over a half of the study samples also included participants from a broad range of BMI categories (n = 15). Finally, although one of the reviewed articles also included men in the total study sample, we only analyzed the data pertaining to women’s experiences. A summary of the key findings from each paper can be found in Additional file 1: Table S1.

### Use of contraception among women in larger bodies

The prevalence and methods of contraception used by women with higher BMIs varied across the reviewed literature (n = 28). While the study findings differed, oral contraceptive pills (OCPs) or male condoms were often reported as the most commonly used method of contraception among women with BMIs classified as ‘overweight’ or ‘obese’ (n = 16). Women with higher BMIs were less likely than women with ‘normal’ BMIs (i.e., < 25 kg/m^2^) to use hormonal contraception (n = 7) and more likely to use LARC or permanent methods (e.g., tubal ligation) (n = 7) [38–47]. To contrast, some researchers found that the use of any method of contraception did not significantly differ by weight status (n = 2), although women with higher BMIs may be more likely than women with lower BMIs to discontinue the use of newly prescribed contraceptives (n = 2) and rely on less effective methods (e.g., withdrawal) or not use contraception at all (n = 4) [38, 40–42, 47–49].

The majority of women undergoing bariatric surgery used contraception [44, 50–60]. Some studies found that contraceptive use improved following bariatric surgery (n = 4), with a greater proportion of women reporting use of highly effective or safe methods of contraception following the procedure, such as LARCs [50–53]. However, the findings from these studies varied, too. While two studies reported initial postsurgical contraception usage rates exceeding 90% [51, 57], a retrospective analysis revealed that fewer than 30% of patient medical charts documented contraceptive use and no new prescriptions were made following bariatric surgery [61]. Nonetheless, a recent systematic review noted that this finding may just reflect poor physician charting practices [59].

Emergency contraception was rarely discussed in the reviewed literature. One article noted that many participants weighed beyond the range of full EC efficacy, but unfortunately, the study did not examine participants’ use of emergency contraception [38]. Similarly, two recent American studies proposed that weight status may influence EC decision-making among women in larger bodies [62, 63]. Following the introduction of European weight-related guidelines on LNG EC labels, the proportion of online UPA purchases made by women with higher BMIs rose significantly in the United States [62], while another study showed that fewer than 30% of American LNG EC users reported a BMI ≥ 26 during the same time period [63].

### Knowledge, attitudes towards and beliefs about contraception

Fewer than half of the reviewed articles provided insight on women’s knowledge, attitudes towards, and beliefs about contraception (n = 11). When making contraceptive decisions, women with higher BMIs may consider ease of use [54] and side effect profile [46, 57, 64]. For instance, a study by Chuang et al. [64] revealed that women with higher BMIs expressed concerns about potential weight gain associated with hormonal methods of contraception. In addition, this study indicated that women in larger bodies did not feel that their body size limited their contraceptive choices and believed that the ability to conceive was largely beyond individual control [64]. Similarly, five studies revealed that women in larger bodies who did not use contraception often believed they were unlikely to become pregnant [42, 56, 57, 59, 65]. Some study participants also attributed their non-use of contraceptives to sexual inactivity, as well as wishing to avoid using contraception or experiencing side effects [56, 57]. In comparison, although the reasons for using EC did not vary by weight [63], the findings from one study demonstrated that some women with higher BMIs made weight-related efficacy considerations when choosing UPA over LNG, explaining that with their weight, “Plan B isn’t as effective” [62].

### Contraceptive (dis)satisfaction among women in larger bodies

Only five studies provided information with regards to women’s (dis)satisfaction with contraception. In general, women who underwent bariatric surgery were content with their method of contraception [51, 55, 58]. However, dissatisfaction was also discussed in three articles, often resulting in contraceptive discontinuation [47, 51, 65]. For example, a study by Callegari et al. [65] determined that relying on withdrawal was associated with previous contraceptive dissatisfaction among women with higher BMIs. Although the reasons for dissatisfaction were not always specified, some women reported contraceptive discontinuation due to changes in their menstrual bleeding patterns [47, 51].

### Contraceptive counseling

While nearly every article advocated for improving contraceptive counseling and care among women with higher BMIs (n = 26), contraceptive counseling was only examined in 16 studies. The majority of the articles discussed counseling in the context of bariatric surgery (n = 10). Many women knew to delay pregnancy following their procedure [50, 54, 56–58]; however, contraceptive counseling appeared to fall short in a number of studies (n = 9) [44, 50, 52, 54, 56–59, 61]. For example, fewer than one third of women who underwent bariatric surgery at three Belgian hospitals reported receiving contraceptive counseling [52]. Moreover, two studies revealed that many women who underwent bariatric surgery did not feel that they received enough information regarding postoperative contraceptive use and family planning [56, 58].

Similarly, findings from three national survey studies suggest that women in larger bodies may receive insufficient contraceptive counseling and care [40, 63, 65]. Numerous studies (n = 8) demonstrated that providing contraceptive counseling positively impacted the contraceptive choices and use of contraceptives among women with higher BMIs [50, 51, 54, 56, 57, 59, 62, 65]. For instance, a study with UPA users revealed that many women with higher BMIs who reported receiving EC counseling were cautioned that LNG would have reduced efficacy due to their weight [62]. Still, the contraceptive counseling that women in larger bodies receive may not always be effective. This was exemplified by an American study where recent EC counseling was reported by a greater proportion of LNG users with a BMI ≥ 26 compared to those with a lower BMI; however, given that LNG EC is believed to have reduced efficacy among women with higher BMIs, the authors suggested that this finding may represent poor provider care or practices [63]. In two other studies, women with higher BMIs were prescribed or accessed COCs despite recommendations or the presence of contraindications [47, 66]. Surprisingly, in a bariatric surgery setting where all women were supposed to have received contraceptive counseling, significantly fewer contraceptive non-users reported receiving counseling compared to women using contraception postoperatively (66.7% vs 95.7%) [57]. In light of these findings, some authors emphasized the need for future research to understand the contraceptive counseling needs and preferences of women in larger bodies [56, 61, 63]. Other authors made clinical practice recommendations (n = 11), including comprehensive contraceptive counseling and monitoring surrounding bariatric surgery [50, 52, 54, 57, 59, 61] as well as the provision of targeted, patient-centered contraceptive counseling and care [40, 51, 56, 63, 65].

### Barriers to contraception

**Fig. 3:**
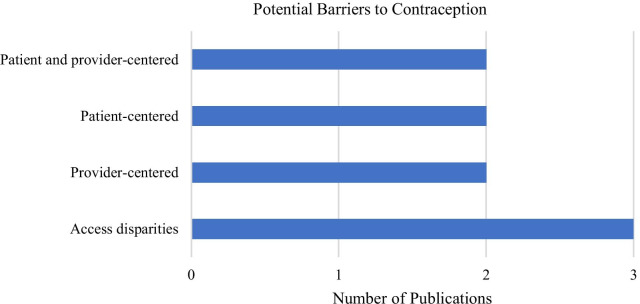
Potential barriers to contraception as outlined in the reviewed literature

While none of the papers explicitly examined the barriers impacting women’s experiences with contraception, some authors discussed potential challenges that women in larger bodies may face when accessing and using contraception (n = 9). This was often framed as access disparities, provider barriers, personal barriers, or a combination thereof (see Fig. 3). Mosher et al. [43] proposed that contraceptive use may differ across weight categories due to disparities in access to care faced by women with higher BMIs. Disparities were often discussed with regards to socioeconomic factors (n = 3), such as income, that may influence access to contraceptive services and continuation of use [43, 47, 65]. Some researchers hypothesized that women with higher BMIs may have unique health care needs that go beyond contraceptive care and thus, health care providers may not prioritize contraceptive counseling or adequately discuss weight-related considerations [41, 46]. In comparison, Becnel et al. [44] suggested that inadequate contraceptive counseling among non-sexually active adolescent girls with higher BMIs may reflect health care provider weight bias. Likewise, three authors proposed that low self-esteem and perceptions of weight stigma may prevent women in larger bodies from accessing contraception or contraceptive care [40–42]. Finally, three articles identified misperceptions and gaps in knowledge among both health care providers or women alike as potential reasons for poor contraceptive planning and use [42, 46, 64].

## Discussion

This scoping review aimed to explore key findings from the literature on women in larger bodies’ experiences with contraception, identify gaps in the literature, and make recommendations for future research. While this review aimed to focus on women’s experiences, the paucity of qualitative data reveals a notable gap in the research. Although women’s voices are rarely represented in the literature, our analysis suggests that women in larger bodies have unmet contraceptive needs. In keeping with our results and the recommendations made by nearly all the authors of the reviewed articles, the current literature underscores the need to improve contraceptive information, counseling, and care for women with higher BMIs.

Women have previously expressed their desire for a close, supportive provider–patient relationship to facilitate open discussions about contraceptive options and aid with informed decision-making [67]. Adding to this, our review highlights the association between contraceptive counseling and the use of highly effective methods of contraception reported by women in larger bodies [50, 51, 54, 56, 59, 62, 65]. Unfortunately, nearly half of the articles (n = 14) revealed that many women with higher BMIs reported inadequate or no contraceptive counseling [40, 44, 47, 50, 52, 54, 56–59, 61, 63, 65, 66]. Moreover, some authors proposed that women in larger bodies may face unique barriers to accessing contraception and contraceptive care, including low self-esteem and perceptions of weight stigma [40–42]. Although women’s perceptions of weight stigma were not examined in any of the reviewed studies, three researchers alluded to the possibility of weight stigma or bias in contraceptive care [41, 42, 44]. This speculation is much in line with previous research in reproductive health care whereby women with higher BMIs have often described negative experiences shaped by disrespectful treatment, presumptuous comments, and the problematization of their bodies [26, 28, 68]. With the ubiquity of weight bias in health care [69, 70] and its relation to health care avoidance among individuals with higher BMIs [71–73], it is crucial that future research explore how women in larger bodies navigate contraceptive care. In doing so, this research can aid in identifying the structural and personal obstacles that may limit women in larger bodies’ access to and engagement with contraceptive counseling and services, gain a better understanding of how women feel about the contraceptive care they receive, and clarify their counseling preferences and needs.

Some studies revealed that women with higher BMIs were more likely than their ‘normal’ BMI counterparts to use less effective or no methods of contraception [38, 40–42]. Moreover, five articles affirmed that women in larger bodies’ contraceptive non-use was often associated with perceived subfertility [42, 56, 57, 59, 65]. Parallel to these findings, perceived infertility has been identified as one of the leading reasons for contraceptive non-use among American women at risk of unintended pregnancy [74]. Although having a BMI ≥ 30 is associated with an elevated risk of fertility issues and miscarriage [75], studies with women who consider themselves to be less likely to become pregnant have yet to attribute this perception to weight or body size [76, 77]. The literature reviewed in this study did not examine why women with higher BMIs felt they were less likely to become pregnant, but this warrants further exploration. Given that women in larger bodies may also experience increased rates of unintended pregnancy [11], future research is needed to examine such perceptions of subfertility. Addressing this gap will provide a better understanding of the factors that contribute to contraceptive discontinuation and non-use among women with higher BMIs, which can thereby inform patient-centered approaches to tailoring sexual health education and contraceptive counseling.

Only two articles in this scoping review focused on emergency contraception, indicating a significant gap in the literature. While the findings from the these studies demonstrate how EC counseling and information about weight-related efficacy may influence women in larger bodies’ decision-making [62, 63], they reveal little about women’s lived experiences. Women have previously described accessing and using EC as stressful or embarrassing, and have reported a range of emotions following usage, including relief, uneasiness, as well as a sense of personal responsibility [78–80]. Considering the stigma associated with both EC [81] and weight [69, 70], women in larger bodies who access and use emergency contraception may be particularly vulnerable to stressful or stigmatizing experiences. Furthermore, while some pharmacists routinely discuss weight-related considerations with EC clients [82], others have reported feeling uncomfortable addressing weight when providing emergency contraceptive counseling [83]. The striking lack of research about women with higher BMIs’ experiences accessing and using EC draws attention to the need for future studies to examine women in larger bodies’ lived experiences with emergency contraception, including how they react to information about weight-related efficacy [62], how they make decisions about EC, and their experiences with emergency contraceptive care.

This study has several limitations. Our focus on recently published empirical papers may have resulted in the exclusion of some important studies that were published prior to 2010. Given that there is little empirical knowledge about women in larger bodies’ experiences with contraception, it may have been beneficial to include a grey literature search to attempt to collect more qualitative data. Following Arksey and O’Malley’s [32] methodology, we did not assess the quality or generalizability of the findings that were reported in the studies we reviewed. Although many of the articles had large sample sizes exceeding 1000 participants, our review only examined research from Western countries. The overrepresentation of white women and bariatric surgery patients, who have unique clinical characteristics and contraceptive needs [59], limits the generalizability of our findings and demonstrates the need for an intersectional approach to future research. In particular, our exclusive focus on women’s experiences calls for further investigation to explore non-binary, transgender, and two-spirit individuals in larger bodies’ experiences with contraception and contraceptive care.

Given that we did not critically appraise the articles included in this review, we did not evaluate other study variables or the impact of potential confounders in the relationship between body size and contraceptive use. For instance, contraceptive choice among American women has previously been associated with a range of socioeconomic factors (e.g., age, race/ethnicity, education, insurance), sexual relationship factors, as well as attitudes towards and experiences with contraceptive methods, pregnancy, and health care providers [84]. Further, due to our broad and inclusive definition of larger bodies, we did not disaggregate the findings based on BMI category, nor did we consider BMI reduction following bariatric surgery. However, among studies that reported postoperative BMI, mean or median BMIs were all above ‘normal’, and often ≥ 30, indicating that many participants would still be considered women in larger bodies [50, 52, 54, 55, 57, 58, 61]. This is consistent with previous research suggesting that the majority of patients do not achieve ‘normal’ BMI after bariatric surgery [85, 86]. Nonetheless, future research should address weight loss and compare women in larger bodies within and across BMI categories to better understand how experiences with contraception may differ by body size, shape, or BMI classification. Finally, most of the literature relied on self-reported data and many authors cited the possibility of bias related to misclassification of BMI [39–43, 49, 54, 62, 63, 65] or self-reported contraceptive behaviors and experiences [41, 46, 48, 56, 57, 60, 63]. To our knowledge, this is the first scoping review to summarize the literature pertaining to women in larger bodies’ experiences with contraception and thus we believe that our findings are valuable despite the aforementioned limitations.

## Conclusions

Women with higher BMIs are a growing population with unique reproductive health needs and risks [6, 75]. As this trend continues, more women of reproductive age will have a height and weight that is considered ‘overweight’ or ‘obese’ [24]. Thus, it is imperative that women in larger bodies have access to highly effective methods of contraception and are provided with patient-centered, non-judgemental contraceptive care to support making informed family planning decisions. This scoping review summarized the empirical literature pertaining to women in larger bodies’ experiences with contraception, revealing that women with higher BMIs may have unmet contraceptive counseling and care needs. We have identified several gaps in the literature and have indicated that additional research, particularly qualitative research, is pressingly needed to better understand, explain, and build upon the current body of knowledge on women in larger bodies’ experiences with contraception. This research will play an integral role in establishing the contraceptive needs of women with higher BMIs and identifying avenues to improve care. Ultimately, information about the lived experiences of women in larger bodies who access and use contraception and contraceptive care may be used in conjunction with the findings from clinical studies to inform updated guidelines for clinical practice. In this way, contraceptive counseling, care, and experiences can be improved for women in larger bodies.

## Supplementary Information

**Additional file 1: Table S1.** Summary of the reviewed literature. This additional table provides an overview of the 29 articles included in our scoping review. The table contains information with respect to the article authors, the country of study, the study design, the total study sample size, as well as key study sample characteristics and research findings pertaining to women in larger bodies’ experiences with contraception.

## Data Availability

The reviewed articles are available in the manuscript and can also be made available upon request.

## Abbreviations

BMI: Body Mass Index
COC: Combined oral contraceptive
Cu-IUD: Copper-bearing intrauterine device
EC: Emergency contraception
IUD: Intrauterine device
IUS: Intrauterine system
LARC: Long-acting reversible contraception
LNG: Levonorgestrel
OCP: Oral contraceptive pill
UPA: Ulipristal acetate

## Footnotes

1 In this paper, we use the term ‘women in larger bodies’ to broadly and inclusively refer to individuals who are categorized as ‘overweight’ or ‘obese’ to avoid the negative connotation and stigma associated with these terms (please see [25–28]). By moving away from the predominant biomedical discourse utilizing BMI classification to indicate body size, we aimed to focus on women’s subjective experiences in their bodies. We also acknowledge that our use of gendered language does not capture the experiences of all contraceptive users in larger bodies.

## References

[1] Ng M, Fleming T, Robinson M, Thomson B, Graetz N, Margono C (2014). Global, regional, and national prevalence of overweight and obesity in children and adults during 1980–2013: a systematic analysis for the global burden of disease study 2013. Lancet.

[2] Mitchell N, Catenacci V, Wyatt HR, Hill JO (2011). Obesity: overview of an epidemic. Psychiatr Clin N Am.

[3] Centers for Disease Control and Prevention. Adolescents who have an overweight classification. National Center for Chronic Disease Prevention and Health Promotion, Division of Nutrition, Physical Activity, and Obesity. Data, Trend and Maps. 2017. https://nccd.cdc.gov/dnpao_dtm/rdPage.aspx?rdReport=DNPAO_DTM.ExploreByTopic&islClass=OWS&islTopic=OWS1&go=GO. Accessed 24 July 2020.

[4] National Center for Health Statistics. Table 26. Normal weight, overweight, and obesity among adults aged 20 and over, by selected characteristics: United States, selected years 1988–1994 through 2013–2016. 2019. https://www.cdc.gov/nchs/hus/contents2018.htm. Accessed 25 July 2020.

[5] World Health Organization. Obesity and overweight. 2020. https://www.who.int/news-room/fact-sheets/detail/obesity-and-overweight. Accessed 27 July 2020.

[6] Kulie T, Slattengren A, Redmer J, Counts H, Eglash A, Schrager S (2011). Obesity and women’s health: an evidence-based review. J Am Board Fam Med.

[7] Kaneshiro B, Jensen JT, Carlson NE, Harvey SM, Nichols MD, Edelman AB (2008). Body mass index and sexual behavior. Obstet Gynecol.

[8] Simmons KB, Edelman AB (2015). Contraception and sexual health in obese women. Best Pract Res Clin Obstet Gynaecol.

[9] Simmons KB, Edelman AB (2016). Hormonal contraception and obesity. Fertil Steril.

[10] Kapp N, Abitbol JL, Mathé H, Scherrer B, Guillard H, Gainer E (2015). Effect of body weight and BMI on the efficacy of levonorgestrel emergency contraception. Contraception.

[11] McKeating A, O’Higgins A, Turner C, McMahon L, Sheehan SR, Turner MJ (2015). The relationship between unplanned pregnancy and maternal body mass index 2009–2012. Eur J Contracept Reprod Health Care.

[12] McNicholas C, Zigler R, Madden T, Jungheim ES (2015). Contraceptive counseling in obese women. Obesity and fertility: a practical guide for clinicians.

[13] Lopez LM, Bernholc A, Chen M, Grey TW, Otterness C, Westhoff C (2016). Hormonal contraceptives for contraception in overweight or obese women. Cochrane Database Sys Rev.

[14] Jatlaoui TC, Curtis KM (2016). Safety and effectiveness data for emergency contraceptive pills among women with obesity: a systematic review. Contraception.

[15] Horton LG, Simmons KB, Curtis KM (2016). Combined hormonal contraceptive use among obese women and risk for cardiovascular events: a systematic review. Contraception.

[16] World Health Organization. Medical eligibility criteria for contraceptive use. 4th ed. 2015. https://apps.who.int/iris/rest/bitstreams/1243459/retrieve. Accessed 15 Jan 2021. 26447268

[17] Edelman A (2009). Contraceptive considerations in obese women. Contraception.

[18] Black A, Guilbert E, Costescu D, Dunn S, Fisher W, Kives S (2015). Canadian contraception consensus (part 1 of 4). J Obstet Gynaecol Can.

[19] Black A, Guilbert E, Costescu D, Dunn S, Fisher W, Kives S (2017). No. 329-Canadian contraception consensus part 4 of 4 chapter 9: combined hormonal contraception. J Obstet Gynaecol Can.

[20] Faculty of Sexual and Reproductive Healthcare (2019). FSRH guideline: overweight, obesity and contraception. BMJ Sex Reprod Health.

[21] Health Canada. Emergency contraceptive pills to carry warnings for reduced effectiveness in women over a certain body weight. 2014. https://www.healthycanadians.gc.ca/recall-alert-rappel-avis/hc-sc/2014/38701a-eng.php. Accessed 29 July 2020.

[22] European Medicines Agency. Levonorgestrel and ulipristal acetate remain suitable emergency contraceptives for all women, regardless of bodyweight. England: European Union; 2014, p. 4. Report No.: 631408. https://www.ema.europa.eu/en/documents/referral/levonorgestrel-ulipristal-remain-suitable-emergency-contraceptives-all-women-regardless-bodyweight_en.pdf. Accessed 29 July 2020.

[23] International Consortium for Emergency Contraception. Emergency contraceptive pills: medical and service delivery guidance. 4th ed. 2018. https://www.cecinfo.org/wp-content/uploads/2018/12/ICEC-guides_FINAL.pdf. Accessed 12 Jan 2021.

[24] Cipriani S, Todisco T, Scavello I, Di Stasi V, Maseroli E, Vignozzi L (2019). Obesity and hormonal contraception: an overview and a clinician’s practical guide. Eat Weight Disord.

[25] Essayli JH, Murakami JM, Wilson RE, Latner JD (2017). The impact of weight labels on body image, internalized weight stigma, affect, perceived health, and intended weight loss behaviors in normal-weight and overweight college women. Am J Health Promot.

[26] LaMarre A, Rice C, Cook K, Friedman M (2020). Fat reproductive justice: navigating the boundaries of reproductive health care. J Soc Issues.

[27] Puhl RM (2020). What words should we use to talk about weight? A systematic review of quantitative and qualitative studies examining preferences for weight-related terminology. Obes Rev.

[28] Bombak AE, McPhail D, Ward P (2016). Reproducing stigma: interpreting “overweight” and “obese” women’s experiences of weight-based discrimination in reproductive healthcare. Soc Sci Med.

[29] Robinson JA, Burke AE (2013). Obesity and hormonal contraceptive efficacy. Womens Health (Lond Engl).

[30] Paulen ME, Zapata LB, Cansino C, Curtis KM, Jamieson DJ (2010). Contraceptive use among women with a history of bariatric surgery: a systematic review. Contraception.

[31] Armstrong C. ACOG guidelines on pregnancy after bariatric surgery. Am Fam Physician. 2010;81(7):905–6. https://www.aafp.org/afp/2010/0401/p905.html. Accessed 29 Mar 2021.

[32] Arksey H, O’Malley L (2005). Scoping studies: towards a methodological framework. Int J Soc Res Methodol.

[33] Anderson J (2012). Whose voice counts? A critical examination of discourses surrounding the body mass index. Fat Stud.

[34] Schoen C, Osborn R, Squires D, Doty MM (2013). Access, affordability, and insurance complexity are often worse in the United States compared to ten other countries. Health Aff.

[35] Papanicolas I, Mossialos E, Gundersen A, Woskie L, Jha AK (2019). Performance of UK National Health Service compared with other high income countries: observational study. BMJ.

[36] Schneider EC, Sarnak DO, Squires D, Shah A, Doty MM. Mirror, mirror 2017: international comparison reflects flaws and opportunities for better U.S. health care. New York: Commonwealth Fund; 2017. http://www.issuelab.org/permalink/download/27698. Accessed 29 Mar 2021.

[37] Kopp W (2019). How Western diet and lifestyle drive the pandemic of obesity and civilization diseases. Diabetes Metab Syndr Obes.

[38] Kohn JE, Lopez PM, Simons HR (2015). Weight and body mass index among female contraceptive clients. Contraception.

[39] Skiba MA, Islam RM, Bell RJ, Davis SR (2019). Hormonal contraceptive use in Australian women: who is using what?. Aust NZ J Obstet Gyn.

[40] Bajos N, Wellings K, Laborde C, Moreau C (2010). Sexuality and obesity, a gender perspective: results from French national random probability survey of sexual behaviours. BMJ.

[41] Chang T, Davis MM, Kusunoki Y, Ela EJ, Hall KS, Barber JS (2015). Sexual behavior and contraceptive use among 18- to 19-year-old adolescent women by weight status: a longitudinal analysis. J Pediatr.

[42] Nguyen BT, Elia JL, Ha CY, Kaneshiro BE (2018). Pregnancy intention and contraceptive use among women by class of obesity: results from the 2006–2010 and 2011–2013 National Survey of Family Growth. Womens Health Issues.

[43] Mosher WD, Lantos H, Burke AE (2018). Obesity and contraceptive use among women 20–44 years of age in the United States: results from the 2011–15 National Survey of Family Growth (NSFG). Contraception.

[44] Becnel JN, Zeller MH, Noll JG, Sarwer DB, Reiter-Purtill J, Michalsky M (2017). Romantic, sexual, and sexual risk behaviours of adolescent females with severe obesity. Pediatr Obes.

[45] Scott-Ram R, Chor J, Bhogireddy V, Keith L, Patel A (2012). Contraceptive choices of overweight and obese women in a publically funded hospital: possible clinical implications. Contraception.

[46] Bhuva K, Kraschnewski JL, Lehman EB, Chuang CH (2017). Does body mass index or weight perception affect contraceptive use?. Contraception.

[47] Sundell M, Ginstman C, Månsson A, Forslund I, Brynhildsen J (2019). Patterns of prescription and discontinuation of contraceptives for Swedish women with obesity and normal-weight women. Eur J Contracept Reprod Health Care.

[48] DeMaria AL, Lugo JM, Rahman M, Pyles RB, Berenson AB (2013). Association between body mass index, sexually transmitted infections, and contraceptive compliance. J Womens Health (Larchmt).

[49] Saito-Tom LY, Soon RA, Harris SC, Salcedo J, Kaneshiro BE (2015). Levonorgestrel intrauterine device use in overweight and obese women. Hawaii J Med Public Health.

[50] Damhof MA, Pierik E, Krens LL, Vermeer M, van Det MJ, van Roon EN (2019). Assessment of contraceptive counseling and contraceptive use in women after bariatric surgery. Obes Surg.

[51] Hillman JB, Miller RJ, Inge TH (2011). Menstrual concerns and intrauterine contraception among adolescent bariatric surgery patients. J Womens Health (Larchmt).

[52] Luyssen J, Jans G, Bogaerts A, Ceulemans D, Matthys C, der Schueren BV (2018). Contraception, menstruation, and sexuality after bariatric surgery: a prospective cohort study. Obes Surg.

[53] Menke MN, King WC, White GE, Gosman GG, Courcoulas AP, Dakin GF (2017). Contraception and conception after bariatric surgery. Obstet Gynecol.

[54] Mody SK, Hacker MR, Dodge LE, Thornton K, Schneider B, Haider S (2011). Contraceptive counseling for women who undergo bariatric surgery. J Womens Health (Larchmt).

[55] Zeller MH, Brown JL, Reiter-Purtill J, Sarwer DB, Black L, Jenkins TM (2019). Sexual behaviors, risks, and sexual health outcomes for adolescent females following bariatric surgery. Surg Obes Relat Dis.

[56] Mengesha BM, Carter JT, Dehlendorf CE, Rodriguez AJ, Steinauer JE (2018). Perioperative pregnancy interval, contraceptive counseling experiences, and contraceptive use in women undergoing bariatric surgery. Am J Obstet Gynecol.

[57] Casas R, Bourjeily G, Vithiananthan S, Tong I (2014). Contraceptive use in women undergoing bariatric surgery. Obes Res Clin Pract.

[58] Ginstman C, Frisk J, Ottosson J, Brynhildsen J (2015). Contraceptive use before and after gastric bypass: a questionnaire study. Obes Surg.

[59] Jäger P, Wolicki A, Spohnholz J, Senkal M (2020). Review: Sex-specific aspects in the bariatric treatment of severely obese women. Int J Environ Res Public Health.

[60] Gosman GG, King WC, Schrope B, Steffen KJ, Strain GW, Courcoulas AP (2010). Reproductive health of women electing bariatric surgery. Fertil Steril.

[61] Mengesha B, Griffin L, Nagle A, Kiley J (2016). Assessment of contraceptive needs in women undergoing bariatric surgery. Contraception.

[62] Cleland K, Wagner B, Smith NK, Trussell J (2020). “My BMI is too high for Plan B.” A changing population of women seeking ulipristal acetate emergency contraception online. Women Health.

[63] Stowers P, Mestad R (2019). Use of levonorgestrel as emergency contraception in overweight women. Obes Res Clin Pract.

[64] Chuang CH, Velott DL, Weisman CS (2010). Exploring knowledge and attitudes related to pregnancy and preconception health in women with chronic medical conditions. Matern Child Health J.

[65] Callegari LS, Nelson KM, Arterburn DE, Prager SW, Schiff MA, Schwarz EB (2014). Factors associated with lack of effective contraception among obese women in the United States. Contraception.

[66] Grossman D, White K, Hopkins K, Amastae J, Shedlin M, Potter JE (2011). Contraindications to combined oral contraceptives among over-the-counter compared with prescription users; 21343758. Obstet Gynecol.

[67] Dehlendorf C, Levy K, Kelley A, Grumbach K, Steinauer J (2013). Women’s preferences for contraceptive counseling and decision making. Contraception.

[68] Parker G, Pausé C (2018). “I’m just a woman having a baby”: negotiating and resisting the problematization of pregnancy fatness. Front Sociol.

[69] Tomiyama AJ, Carr D, Granberg EM, Major B, Robinson E, Sutin AR (2018). How and why weight stigma drives the obesity ‘epidemic’ and harms health. BMC Med.

[70] Phelan SM, Burgess DJ, Yeazel MW, Hellerstedt WL, Griffin JM, van Ryn M (2015). Impact of weight bias and stigma on quality of care and outcomes for patients with obesity. Obes Rev.

[71] Puhl R, Peterson JL, Luedicke J (2013). Motivating or stigmatizing? Public perceptions of weight-related language used by health providers. Int J Obes.

[72] Forhan M, Risdon C, Solomon P (2013). Contributors to patient engagement in primary health care: perceptions of patients with obesity. Prim Health Care Res Dev.

[73] McGuigan RD, Wilkinson JM (2015). Obesity and healthcare avoidance: a systematic review. AIMS Public Health.

[74] Frederiksen BN, Ahrens K (2020). Understanding the extent of contraceptive non-use among women at risk of unintended pregnancy, National Survey of Family Growth 2011–2017. Contraception: X.

[75] Lash MM, Armstrong A (2009). Impact of obesity on women’s health. Fertil Steril.

[76] Polis CB, Zabin LS (2012). Missed conceptions or misconceptions: perceived infertility among unmarried young adults in the United States. Perspect Sex Reprod Health.

[77] Gemmill A (2018). Perceived subfecundity and contraceptive use among young adult U.S. women. Perspect Sex Reprod Health.

[78] Shoveller J, Chabot C, Soon JA, Levine M (2007). Identifying barriers to emergency contraception use among young women from various sociocultural groups in British Columbia, Canada. Perspect Sex Reprod Health.

[79] Williamson LM, Buston K, Sweeting H (2009). Young women’s perceptions of pregnancy risk and use of emergency contraception: findings from a qualitative study. Contraception.

[80] Gainer E, Blum J, Toverud E-L, Portugal N, Tyden T, Nesheim B-I (2003). Bringing emergency contraception over the counter: experiences of nonprescription users in France, Norway, Sweden and Portugal. Contraception.

[81] Eastham R, Milligan C, Limmer M (2020). Qualitative findings about stigma as a barrier to contraception use: the case of emergency hormonal contraception in Britain and implications for future contraceptive interventions. Eur J Contracept Reprod Health Care.

[82] Chaumont A, Foster AM (2017). The not so over-the-counter status of emergency contraception in Ontario: a mixed methods study with pharmacists. FACETS.

[83] Wong K, Hum S, McCarthy L, Dunn S (2017). Beyond Plan B: a qualitative study of Canadian pharmacists’ emergency contraception counselling practices. J Obstet Gynaecol Can.

[84] Frost JJ, Darroch JE (2008). Factors associated with contraceptive choice and inconsistent method use, United States, 2004. Perspect Sex Reprod Health.

[85] Lager CJ, Esfandiari NH, Subauste AR, Kraftson AT, Brown MB, Cassidy RB (2017). Milestone weight loss goals (weight normalization and remission of obesity) after gastric bypass surgery: long-term results from the University of Michigan. Obes Surg.

[86] Cadena-Obando D, Ramírez-Rentería C, Ferreira-Hermosillo A, Albarrán-Sanchez A, Sosa-Eroza E, Molina-Ayala M (2020). Are there really any predictive factors for a successful weight loss after bariatric surgery?. BMC Endocr Disord.

