# Supplementary material for: Women in larger bodies’ experiences with contraception: a scoping review
Source: Reprod Health. 2021 Apr 29;18:89. doi: 10.1186/s12978-021-01139-2 (PMC8082612; doi:10.1186/s12978-021-01139-2)
Supplement: Supplementary file 1 — Additional file 1: Table S1. Summary of the reviewed literature. This additional table provides an overview of the 29 articles included in our scoping review. The table contains information with respect to the article authors, the country of study, the study design, the total study sample size, as well as key study sample characteristics and research findings pertaining to women in larger bodies’ experiences with contraception. [file 12978_2021_1139_MOESM1_ESM.docx]

Table S1. Summary of the reviewed studies

| **Authors** | **Country** | **Study design** | **Total study sample size** | **Key sample characteristics** | **Key findings** |
| --- | --- | --- | --- | --- | --- |
| **Bajos et al. (2010)** | France | Quantitative (Cross-sectional) | 10 170 | National sample of 5535 women (21% with BMI 25-29.9, 9% with BMI > 30). | The use of any contraceptive was similar across BMI categories. Compared to women with lower BMIs, women with higher BMIs were less likely to use condoms and OCPs, and more likely to use withdrawal. Women with a BMI > 30 were also less likely to have seen a physician for contraceptive care in the last year, which the authors suggest may reflect internalized weight stigma. |
| **Becnel et al. (2017)** | United States | Quantitative (Prospective longitudinal observational cohort) | 294 | Three group clinical sample of adolescent girls aged 13-18. Two clinical groups of girls with a BMI > 40: 108 bariatric surgery patients and 68 lifestyle intervention patients. The third group included 118 ‘normal weight’ comparisons. | Both clinical groups were less likely to report unprotected sex than the ‘normal weight’ control group; however, surgical patients were more likely to use contraception consistently and non-surgical patients were less likely to use hormonal contraception. While the majority of sexually active adolescents reported contraceptive counseling from their doctors, 25% of non-sexually active girls did not receive any information. |
| **Bhuva et al. (2017)** | United States | Quantitative (Cross-sectional) | 987 | Privately insured women between the ages of 18-40 at risk of unintended pregnancy (22.4% with BMI 25-29.9, 19.7% with BMI > 30). | Compared to women with lower BMIs, those with higher BMIs were more likely to use LARCs, less effective (e.g., non-prescription) or no methods of contraception than non-LARC prescription methods. Odds of LARC use remained significant after adjusting for weight perception. The authors proposed that women with higher BMIs may be concerned with weight gain associated with non-LARC prescription contraceptives (e.g., OCPs) and discussed health care provider barriers that may impact contraceptive care. |
| **Callegari et al. (2014)** | United States | Quantitative (Cross-sectional) | 1345 | National sample of women aged 20-44 with a BMI > 30 at risk of unintended pregnancy. | While OCPs were the most commonly reported method of contraception, contraceptive non-use was reported by over 20% of women, which was associated with perceived subfertility. Prescription method users were significantly more likely than any other group to report contraceptive counseling, but overall, fewer than half of the study sample reported discussing contraception with a health care provider within the past year. This may reflect barriers in access to care. |
| **Casas et al. (2014)** | United States | Quantitative (Cross-sectional) | 35 | Heterosexual women between the ages of 18-44 registered at a bariatric surgery clinic. Mean preoperative BMI of 53.8 ± 9.3 and mean postoperative BMI of 33.5 ± 10. | Women’s contraceptive use decreased from over 90% after surgery to only two thirds at the time of the survey. OCPs, condoms, and IUDs were the most commonly use method pre- and postoperatively. The vast majority of women reported receiving contraceptive counseling (85.7%) and knew to avoid pregnancy following surgery (80%), a greater proportion of which was reported by current contraceptive users compared to non-users. Side effects, perceived subfertility, and current sexual inactivity were identified as reasons for contraceptive non-use. |
| **Chang et al. (2015)** | United States | Quantitative (Prospective longitudinal cohort) | 900 | Heterosexual women between the ages of 18-19 (24% with BMI 25-29.9, 21% with BMI > 30). | Sexually active women with higher BMIs were less likely to use any method of contraception compared to their ‘normal’ BMI counterparts. Contraception was used less consistently by sexually active women with higher BMIs, with OCPs being used particularly less frequently when compared to women with lower BMIs. The authors suggest that young women with higher BMIs may face personal, provider, and system level barriers to contraception. |
| **Chuang et al. (2010)** | United States | Qualitative (Focus groups) | 72 | Forty women with a BMI > 30 separated into five focus groups, representing the ‘obesity’ sub-sample of a study of women aged 20-45 with chronic conditions. | Women with higher BMIs did not believe their weight limited their contraceptive options. Participants with higher BMIs were also concerned about potential weight gain associated with certain methods of contraception and expressed low perceived control over their ability to become pregnant. |
| **Cleland et al. (2020)** | United States | Quantitative (Cross-sectional) | 8009 | Women purchasing UPA through an online pharmacy (21.5% with BMI 25-29.9, 33.1% with BMI > 30). | The proportion of women with a BMI > 30 purchasing UPA increased significantly following the European LNG label change, while purchases from women with a BMI of 25-29.9 did not change. Many women who provided open-ended responses indicated that their BMI or weight influenced their decision to purchase UPA, several of whom reported that their health care provider counseled them about weight-related efficacy. |
| **Damhof et al. (2019)** | Netherlands | Quantitative (Cross-sectional) | 230 | Multi-site sample of women aged 18-45 who underwent bariatric surgery within the last five months. Mean preoperative BMI of 43.9 and mean current BMI of 36.9. | Three quarters of all women reported postoperative use of contraception – 60% reported use of a ‘safe’ method (e.g., LARC, injections) and 16% reported use of an ‘unsafe’ method (i.e., OCPs). Use of ‘safe’ methods increased after surgery and use of ‘unsafe’ methods decreased. Although those who received contraceptive counseling had greater odds of ‘safe’ contraceptive use and most women knew to avoid pregnancy following bariatric surgery, over one third of participants did not receive any contraceptive counseling. |
| **DeMaria et al. (2013)** | United States | Quantitative (Prospective longitudinal cohort) | 1015 | Multi-site sample of low-income girls and women aged 16-24 (30% with BMI 25-29.9, 28.4% with BMI > 30). | The majority of contraceptive behaviors did not differ by BMI category; however, women with a BMI of 25-29.9 were more likely to report contraceptive non-use at the three-month follow-up compared to those with a ‘normal’ BMI. |
| **Ginstman et al. (2015)** | Sweden | Quantitative (Cross-sectional) | 563 | Women aged 22-43 who underwent bariatric surgery in 2010. Preoperative BMI ranged from 29.7-73.5 (mean 43.6) and current BMI ranged from 18.4-64.1 (mean 27.1). | Approximately one third of women reported no contraceptive use in the first year following surgery. The IUD and IUS were the most frequently used methods pre- and post-surgery, and most women using contraception were satisfied with their current method (82%). While the majority of women received information about delaying pregnancy, nearly one fifth wished they had received more contraceptive counseling and others did not receive any counseling. |
| **Gosman et al. (2010)** | United States | Quantitative (Retrospective and cross-sectional) | 1538 | Sub-sample of 660 women of reproductive age (aged 18-44) from a study with women undergoing bariatric surgery (mean BMI 47.2, ranging from 33.8-87.3). | Approximately half of women reported contraceptive use in the year before bariatric surgery. Among recent contraceptive users at risk for pregnancy, nearly 75% reported always using contraception. Condoms, OCPs, and withdrawal were the most commonly reported methods used. |
| **Grossman et al. (2011)** | United States | Quantitative (Prospective cohort) | 1015 | Women aged 18-44 living near the Mexican border who accessed COCs: 514 from Texas clinics and 501 over the counter in Mexico (34.8% with BMI 25-29.9 and 33% with BMI > 30). | Women with BMI > 30 had higher odds of contraindication for COCs. Although the prevalence of contraindications was significantly higher among women who obtained COCs over-the-counter in Mexico, nearly 20% of women who accessed COCs in Texas had contraindications, suggesting insufficient contraceptive screening and counseling. |
| **Hillman et al. (2011)** | United States | Quantitative (Historic cohort) | 25 | Adolescent girls who received contraceptive counseling prior to bariatric surgery (mean BMI of 51.4 ± 6.3, mean age 17.4). | Approximately half of participants had a history of hormonal contraceptive use, including OCPs, vaginal rings, and contraceptive patches. Nearly all participants decided to have an LNG-IUD placed at the time of bariatric surgery. The IUD was well-tolerated by the majority of users as reflected by high voluntary continued use at six-month follow up. |
| **Jäger et al. (2020)** | Germany | Review  (Systematic) | 106 articles | Eight articles in the ‘Contraception’ category in a review focused on sex-related differences among bariatric surgery patients. | The literature suggests that women often receive insufficient contraceptive counseling from bariatric surgeons; however, women who receive counseling tend to use more effective methods. The findings highlight the importance of high-quality contraceptive counseling provided by bariatric surgeons. |
| **Kohn et al. (2015)** | United States | Quantitative (Cross-sectional) | 147 336 | Multi-site sample of girls and women aged 15-44 (23.9% with BMI 25-29.9 and 22.2% with BMI > 30). | When compared to women with lower BMIs, women with BMI > 30 had greater odds of using the most effective (e.g., LARC) and least effective methods of contraception (e.g., withdrawal), as well as reduced odds of hormonal contraceptive use. |
| **Luyssen et al. (2018)** | Belgium | Quantitative (Prospective cohort) | 71 | Multi-site sample of women aged 18-43 undergoing bariatric surgery. Preoperative BMI ranged from 34.4-64.5 (mean 42.0) and 12-month postoperative BMI ranged from 21.9-36.3 (mean 26.7). | LARC use increased following surgery, whereas short-acting hormonal contraceptive use decreased. However, compared to decreased use of OCPs, non-oral methods including IUDs, implants, injections, vaginal rings, condoms, and sterilization increased postoperatively. Approximately one third of women in this study reported receiving contraceptive counseling. |
| **Mengesha et al. (2016)** | United States | Quantitative (Retrospective cohort) | 1012 | Medical charts of women aged 18-45 who were evaluated for bariatric surgery. Preoperative BMI ranged from 32-96 (mean 48.5), and 12-month postoperative BMI ranged from 21-78 (mean 34.4). | Fewer than 30% of charts reviewed indicated current or planned contraceptive use, with OCPs and sterilization being the most frequently documented methods. Family planning counseling was documented in seven charts (0.7%), and two of these women requested gynecology referrals for contraceptive management. |
| **Mengesha et al. (2018)** | United States | Quantitative (Cross-sectional) | 363 | Women aged 18-45 who underwent bariatric surgery within the last two years. No data on BMI. | Two thirds of women reported ever using contraception in the first year following surgery, with the most commonly reported methods being condoms, OCPs, and IUD. Contraceptive use was associated with receiving perioperative family planning counseling. Although 74% of women reported discussions about pregnancy and contraception, nearly half of these women desired more information and counseling. The majority of contraceptive counseling was provided by bariatric surgeons, although some patients wished they had access to a women’s health specialist at the bariatric care clinic. In comparison, contraceptive non-use was often attributed to sexual inactivity, personal choice, and perceived infertility. |
| **Menke et al. (2017)** | United States | Quantitative (Prospective longitudinal cohort) | 710 | Multi-site sample of women aged 18-44 undergoing bariatric surgery. Preoperative BMI ranged from 42.4-51.4 (median 46.3). | Contraception use increased in the first year after surgery and the use of IUDs and sterilization continued to increase in the seven years of follow-up. Nonetheless, over 40% of women reported having unprotected intercourse while not trying to become pregnant in the first year following surgery. |
| **Mody et al. (2011)** | United States | Quantitative (Cross-sectional) | 69 | Clinical sample of women aged 26-40 who underwent bariatric surgery. Preoperative BMI ranged from 42.9-52.0 (median 46.4), postoperative BMI ranged from 33.9-43.4 (median 39.0). | Condoms were the most frequently reported method used both before and after surgery, followed by OCPs or no contraception. The most commonly reported reason for method choice was ease of use (38.7%). Although nearly all women knew they needed to avoid pregnancy after surgery, only about 20% of women were referred to a specialist for contraceptive counseling. These women were more likely to use IUDs following surgery. |
| **Mosher et al. (2018)** | United States | Quantitative (Cross-sectional) | 6562 | National sample of women aged 15-44 at risk of unintended pregnancy (27.3% with BMI 25-29.9, 30.5% with BMI > 30). | With increasing BMI, women had greater odds of sterilization and reduced odds of hormonal method use. Similarly, women with higher BMIs were more likely to use IUD or implant and less likely to use hormonal contraceptives when compared to women with lower BMIs. The authors proposed that differences in contraceptive use across BMI categories may be due to access barriers faced by women with higher BMIs. |
| **Nguyen et al. (2018)** | United States | Quantitative (Cross-sectional) | 9848 | National sample of women aged 20-44 at risk of unintended pregnancy (27.7% with BMI 25-29.9, 33.4% with BMI > 30). | Sterilization was the most frequently reported method of contraception. In general, women with BMI > 35 had greater odds of contraceptive non-use compared to those with ‘normal’ BMI, which may be associated with reported perceived sub-fertility and barriers to contraception. Finally, women with BMI 35-39.9 had greater odds of LARC use or sterilization compared to short-acting hormonal methods. |
| **Saito-Tom et al. (2015)** | United States | Quantitative (Retrospective cohort) | 149 | Multi-site medical chart sample of women who had an LNG-IUD inserted and followed-up after one year (30% with BMI 25-29.9, 33% with BMI > 30). | Women with higher BMIs were more likely to have their LNG-IUD inserted by a resident physician. Compared to women with ‘normal’ BMIs, women with BMI 25-29.9 had higher odds of continued IUD use 12-months following insertion, whereas the odds were lower for women with BMI > 30. However, the odds did not reach statistical significance. |
| **Scott-Ram et al. (2012)** | United States | Quantitative (Retrospective cohort) | 7262 | Medical charts of girls and women aged 16-38 with contraceptive choice documented after post-abortion contraceptive counseling (29.5% with BMI 25-29.9, 34.5% with BMI > 30). | Women with higher BMIs were more likely to choose sterilization, IUD, or the contraceptive ring compared to women with lower BMIs, and less likely to choose the contraceptive injection or patch. Selection of condoms, OCPs, and the contraceptive implant did not vary by BMI. |
| **Skiba et al. (2019)** | Australia | Quantitative (Cross-sectional) | 6600 | Multi-region sample of women aged 18-39 (22.2% with BMI 25-29.9, 24.3% with BMI > 30). | Women with a BMI > 30 were less likely to use hormonal contraception compared to women with ‘normal’ BMIs, but more likely to use a hormonal implant or injection than a hormonal IUS. In addition, women with higher BMIs were more likely to use LARCs or the contraceptive injection compared to COCs. |
| **Stowers & Mestad (2019)** | United States | Quantitative (Cross-sectional) | 5699 | National sample of women aged 18-44 (51.6% who reported a BMI > 26). | Recent LNG use was more frequently reported by women with lower BMIs compared to those with higher BMIs. LNG users with a BMI > 26 were more likely to have received EC counseling than those with a BMI < 26 (40.2% vs 18.3%), which may suggest poor provider knowledge or practices. |
| **Sundell et al. (2019)** | Sweden | Quantitative (Retrospective cohort) | 1115 | Multi-site medical chart sample of 371 women aged 18-40 with BMI 30-50 (median BMI 33) and 744 case-matched women with BMI 19-25 (median BMI 22). | Use of progestin-only methods (e.g., OCPs, IUS, injection, implant) and the Cu-IUD were more common among women with higher BMIs compared to those with lower BMIs. One fifth of women with higher BMIs were prescribed COCs despite national and European recommendations. Among incident users, women with higher BMIs were more likely to discontinue their new contraceptive within the first year of the study period. The most commonly reported reason for discontinuation was menstrual disruption. |
| **Zeller et al. (2019)** | United States | Quantitative  (Prospective cohort) | 179 | Multi-site sample of girls aged 13-19 undergoing bariatric surgery (n=111) and non-surgical comparisons (n=68). Mean baseline BMI 50.99 ± 8.42 and 46.47 ± 5.83, respectively. Mean two-year follow-up BMI was 39.27± 10.08 for surgical patients and 48.56 ± 9.84 for non-surgical patients. | At two-year follow up, a greater proportion of girls who underwent bariatric surgery reported the use of any method of contraception at last sexual intercourse and felt confident that their contraception would prevent pregnancy compared to non-surgical patients. |
